# Methodology of the third British National Survey of Sexual Attitudes and Lifestyles (Natsal-3)

**DOI:** 10.1136/sextrans-2013-051359

**Published:** 2013-11-26

**Authors:** Bob Erens, Andrew Phelps, Soazig Clifton, Catherine H Mercer, Clare Tanton, David Hussey, Pam Sonnenberg, Wendy Macdowall, Nigel Field, Jessica Datta, Kirstin Mitchell, Andrew J Copas, Kaye Wellings, Anne M Johnson

**Affiliations:** 1 Research Department of Infection & Population Health, University College London, London, UK; 2 National Centre for Social Research, London, UK; 3 Department of Social and Environmental Health Research, London School of Hygiene & Tropical Medicine, London, UK; 4 Department of Health Services Research and Policy, London School of Hygiene & Tropical Medicine, London, UK

**Keywords:** SEXUAL BEHAVIOUR, SEXUAL HEALTH, SOCIAL SCIENCE, STATISTICS

## Abstract

**Background:**

Data from the first two National Surveys of Sexual Attitudes and Lifestyles, carried out in 1990–1991 (Natsal-1) and 1999–2001 (Natsal-2), have been extensively used to inform sexual health policy in Britain over the past two decades. Natsal-3 was carried out from September 2010 to August 2012 in order to provide up-to-date measures of sexual lifestyles and to extend the scope of the previous studies by including an older age group (up to 74 years), an extended range of topics and biological measures.

**Methods:**

We describe the methods used in Natsal-3, which surveyed the general population in Britain aged 16–74 years (with oversampling of younger adults aged 16–34 years).

**Results:**

Overall, 15 162 interviews were completed, with a response rate of 57.7% and a cooperation rate of 65.8%. The response rate for the boost sample of ages 16–34 years was 64.8%, only marginally lower than the 65.4% achieved for Natsal-2, which surveyed a similar age range (16–44). The data were weighted by age, gender and region to reduce possible bias. Comparisons with census data show the weighted sample to provide good representation on a range of respondent characteristics. The interview involved a combination of face-to-face and self-completion components, both carried out on computer. Urine samples from 4550 sexually-experienced participants aged 16–44 years were tested for a range of STIs. Saliva samples from 4128 participants aged 18–74 years were tested for testosterone.

**Conclusions:**

Natsal-3 provides a high quality dataset that can be used to examine trends in sexual attitudes and behaviours over the past 20 years.

## Background

Improving sexual and reproductive health remains a high priority in Britain.^1^ ^2^ Findings from the two previous National Surveys of Sexual Attitudes and Lifestyles (Natsal) have been widely used to inform sexual health policy. Natsal-1 (1990–1991) interviewed a probability sample of 18 876 adults aged 16–59 years while Natsal-2 (1999–2001) interviewed 12 110 adults aged 16–44 years. Natsal-1 and Natsal-2 demonstrated the feasibility of carrying out a survey on sexual behaviour and lifestyles in the general population in Britain. Extensive development work on language and question wording, questionnaire format, the collection of urine samples, psychometrically validated measures of particular outcomes (eg, unplanned pregnancy) and, in Natsal-2, the use of computer-assisted self-interviewing (CASI) ensured optimal data quality.^3^

Natsal-3 was funded by grants from the Medical Research Council and the Wellcome Trust with contributions from the Economic and Social Research Council and the Department of Health. Natsal-3 aimed to interview a representative sample of 15 000 men and women aged 16–74 years resident in Britain, using computer-assisted methods, in order to obtain behavioural, attitudinal and biological data and explore their relationships with a range of sexual and reproductive health outcomes. The age range was extended in Natsal-3 to 74 years out of recognition that many individuals continue to be sexually active into their later years and that sexual health issues affect older as well as younger people, and because of a lack of survey data available for this increasing segment of the British population. Natsal-3 psychometrically validated a new measure of sexual function,^4^ and tested a range of sexually transmitted infections (STIs) including *Chlamydia trachomatis*, *Neisseria gonorrhoeae*, type-specific human papillomavirus, HIV antibody and *Mycoplasma genitalium.* An assay for measuring testosterone in saliva was also validated for the survey.

Natsal-3 was granted ethical approval from the Oxford A NHS Research Ethics Committee (reference: 09/H0604/27).

This paper describes the survey methods used in Natsal-3, covering sample design, questionnaire content, data collection, response rates, weighting and the representativeness of the data.

## Sample design

The sample size for Natsal-3 was calculated to provide robust estimation of major parameters (eg, the number of sexual partners over defined time periods, age at first intercourse, same-sex experience) and to detect significant changes in key behaviours when comparing the three Natsals. Taking into account the complex sample design, the target sample size was set at 15 000. Younger adults aged 16–34 years were ‘boosted’ to constitute approximately half of the sample in order to provide sufficient statistical power for exploring behaviours among those at the highest risk of a range of sexual health outcomes. The aim was to achieve approximately equal numbers of around 1900 (with an effective sample size of around 1400 due to clustering) in 5-year age bands up to age 34, declining to around 800 (effective sample size of about 600) for participants aged 65–74 years.

As for Natsal-1 and Natsal-2, the sample frame was the (small users) Postcode Address File (PAF), a regularly updated list of all addresses in the country. The PAF excludes the homeless, and the survey excluded residents living in institutions, so Natsal-3 is representative of individuals living in private residential households. Since the PAF lists only addresses, and provides no information about residents, a sampling procedure is required to select one resident at the sampled address. As a result, individuals in large households have a lower chance of selection than those in smaller households, and it becomes essential to weight the data to take account of different selection probabilities.

Natsal-3 involved a multi-stage, clustered and stratified probability sample design, with postcode sectors selected as the primary sampling units (PSUs), addresses within them selected at the second stage and one eligible person selected at the final stage. Before selection, using data from the 2001 census, the PSUs were stratified—by region, population density, the proportion of the population aged under 60 and the proportion of households with a head in a non-manual occupation—in order to maximise precision of the sample and to ensure that different strata were correctly represented. The sectors were selected systematically, with each sector being given a probability of selection proportional to its total number of addresses.

Overall, 1727 sectors were selected. Fieldwork was split into eight ‘waves’, with each wave issued roughly every quarter over the 2 years of data collection. Within each sector, selected addresses were randomly allocated to: the ‘core’ sample which screened for individuals aged 16–74 years; the ‘boost 1’ sample which screened for those aged 16–34 years; or the ‘boost 2’ sample which screened for those aged 16–29 years. At each address where contact was made, one person was selected at random using a Kish grid technique.^5^ A table showing the number of PSUs and addresses issued per wave is provided as an online supplementary appendix.

## The questionnaire

The Natsal-3 questionnaire was similar to those used in Natsal-1 and Natsal-2. Natsal-3 involved a combination of face-to-face interview using computer-assisted personal interviewing (CAPI), and self-completion format using CASI. A description of the development phase of the Natsal-1 instrument covers questionnaire wording, confidentiality, reliability, validity and so on, as well as the piloting work that preceded the 1990 survey.^6^ ^7^ Details of the development of the Natsal-2 questionnaire are also available, along with results from an experiment that compared reports of sexual behaviour using paper and pencil (PAPI, which was used in Natsal-1) versus CASI methods (used in Natsal-2 and Natsal-3).^8^ ^9^

Natsal-3 included new questions on topics that were relevant to the older age range included in the survey or to cover new areas of interest (including on health conditions or medications taken that might affect a person's sex life, use of Viagra, menopause and use of HRT, sexual function and non-volitional sex). Natsal-3 questionnaire topics are shown in box 1. The questionnaire underwent thorough development work, including cognitive testing and two large-scale pilots.^10^ ^11^

Box 1 **Natsal-3 questionnaire content**

- General health, health conditions, medications taken, medical procedures (that may affect a person's sex life)
- Family when growing up
- Learning about sex
- First heterosexual experience
- Contraception used
- Periods, menopause and use of hormone replacement therapy
- Experience of different heterosexual practices (vaginal, oral and anal intercourse)*
- Opposite-sex sex in the last 4 weeks and condom use*
- Same-sex sexual experiences (types of sexual practices, sex in last 4 weeks)
- Number of opposite-sex partners in different time periods (lifetime, 5 years, 1 year, 3 months)*
- Number of same-sex partners in different time periods*
- Details of most recent partners*
- Having sex with people from other countries and while abroad*
- Non-volitional sex*
- Paying for sex*
- Family formation, pregnancy history and unplanned pregnancy*
- Fertility intentions and infertility*
- STI diagnoses and clinic attendance, HPV vaccination and cervical screening*
- Circumcision*
- HIV testing*
- Sexual function and satisfaction*
- Use of Viagra*
- Use of recreational drugs*
- Screen for depressive symptoms*
- Attitudes to different kinds of relationship
- Socio-demographics

*Asked in CASI.

Participants who reported no sexual experience of any kind were not routed into the CASI (142 men, 150 women), and those who reported no heterosexual sex (defined as vaginal, oral or anal sex) and no same-sex sexual experience involving genital contact were given a shortened version of the CASI (277 men, 310 women).

CASI, computer-assisted self-interviewing; HPV, human papillomavirus; STI, sexually transmitted infection.

Interviews took place in participants’ own homes. Interviewers were present in the room while participants completed the CASI part of the questionnaire, but they were not allowed to view responses. At the end of the CASI, answers were ‘locked’ in the laptop and could not be accessed by interviewers. Median interview length was 53 min.

## Recruitment and response rate

Sampled addresses were sent an advance letter and leaflet giving background information about Natsal-3. Soon after, interviewers personally visited each address, established whether any residents were within the eligible age range, and randomly selected one person. The survey was then fully explained to the selected person, a more detailed information leaflet was provided and verbal consent was sought for the interview.

Table 1 shows address outcomes and response rates overall and by sample type. Sampling from PAF means that some selected addresses are out of scope (eg, because they are non-residential). Also, since we were including only individuals aged 16–74 years (16–34 years at boost addresses), a number of selected addresses did not include anyone within this range. After excluding these addresses, there were 27 503 potentially eligible addresses. No information was obtained at 4143 of these addresses (eg, because no contact was made). Recommended practice is to use the best evidence available for estimating the proportion of ineligibles at addresses where eligibility is unknown.^12^ Assuming the percentage of ineligibles at the unknown addresses is the same as for the known addresses, a further 1229 addresses were estimated as ineligible. Interviews were completed with 15 162 participants at the 26 274 estimated eligible addresses, giving an overall response rate of 57.7% (this follows the formula for calculating the American Association for Public Opinion Research (AAPOR) Response Rate 3).^13^ Participants received a £15 gift voucher as a token of appreciation. Response can also be shown as a range with a lower limit (by assuming all addresses where eligibility is unknown are eligible) and an upper limit (all unknown addresses are assumed to be ineligible). Based on these assumptions, Natsal-3 response ranges from 55.1% (formula for AAPOR RR1) to 64.9% (formula for AAPOR RR5). Based on all eligible units contacted, the cooperation rate (AAPOR formula for Cooperation Rate 2) for Natsal-3 was 65.8%.

**Table 1:** Response rate for Natsal-3 core and boost samples

	All		Core (16–74)		Boost 1 (16–34)		Boost 2 (16–29)	
	N	%	N	%	N	%	N	%
Sampled addresses	59 412	100	24 924	100	18 537	100	15 951	100
Known ineligible addresses								
Vacant/derelict	3137	5.3	1620	6.5	828	4.5	689	4.3
Non-residential	710	1.2	310	1.2	203	1.1	197	1.2
Not traced/built/other	177	0.3	92	0.4	39	0.2	46	0.3
Not eligible age range	27 885	46.9	3613	14.5	12 438	67.1	11 834	74.2
Total known ineligibles	31 909	53.7	5635	22.6	13 508	72.9	12 766	80.0
Unknown eligibility								
No contact	1056	1.8	698	2.8	206	1.1	152	1.0
All information refused	2501	4.2	2048	8.2	291	1.6	162	1.0
Other	586	1.0	418	1.7	106	0.6	62	0.4
Total unknown eligibility	4143	7.0	3164	12.7	603	3.3	376	2.4
Estimated ineligible	1229		525		418		286	
Total estimated eligible addresses	26 274	100	18 764	100	4611	100	2899	100
No interview								
No contact with selected person	327	1.2	190	1.0	86	1.9	51	1.8
Refused (including proxy refusal)	6343	24.1	4668	24.9	1049	22.8	626	21.6
Other reason	1528	5.8	1043	5.6	303	6.6	182	6.3
No information about address	2914	11.1	2639	14.1	185	4.0	90	3.1
Total unproductive	11 112	42.3	8540	45.5	1623	35.2	949	32.7
Completed interviews	15 162	57.7	10 224	54.5	2988	64.8	1950	67.3

## Collecting and testing the urine samples for STIs

Men and women aged 16–44 years, except those with no sexual experience, were eligible to provide a urine sample. At the end of the interview, interviewers gave a subsample of participants a verbal explanation and a leaflet describing the purpose of the urine tests and what was involved. It was explained that the tests would be anonymised, and that participants would not be given their individual results. Written signed consent was obtained for collecting and testing the sample, with separate consent for storage of any remaining urine for future measurement of other (unspecified) pathogens. The full protocol for urine sample collection, the reasons for not returning results and the anonymisation and data linkage procedures are described elsewhere.^14^ ^15^

With a target of 5000 samples, and assuming a 70% response rate, all eligible 16–24-year-olds, all men aged 16–44 years who reported having sex with another man in the last 5 years, and a randomly selected 85% of other eligible participants aged 25–44 years (covering all PSUs) were asked to provide a sample. Of the 8047 participants who reported ever having sex and were invited to provide a urine sample, 4828 agreed (60.0%). After taking account of insufficient samples, mislabelling or unrecorded consent, the number of useable urine samples was 4550 (56.5% of eligible participants). (*All* 16- and 17-year-olds were asked to provide a sample, regardless of their sexual experience, so as not to inadvertently alert others in the household as to the young person's sexual experience.)

Up to 5 mL of urine was collected in the sterile plastic FirstBurst device, designed to catch the first part of the stream, and which yields a specimen with a sixfold higher *C trachomatis* organism load than the regular urine cup.^16^ Samples were posted by the interviewer, on the same day, to the CPA accredited (Clinical Pathology Accreditation (UK)) laboratories at Public Health England Colindale. Participants who provided a sample were given an additional £5 gift voucher.

Upon receipt in the laboratory, specimens were divided into aliquots ahead of their respective testing procedures. Further details of sample preparation, testing and quality assurance are available elsewhere.^15^ We initially tested for *C trachomatis*, *N gonorrhoeae* (GC), *M genitalium*, human papillomavirus types and HIV antibody. With consent, remaining urine was stored at −80°C, and subsequently tested for *Trichomonas vaginalis*, with residual material stored for future testing.

## Collecting and testing the saliva samples for testosterone

Participants aged 18–74 (except those who regularly worked night shifts) were eligible to provide a saliva sample to be tested for testosterone. At the end of the interview (and after urine sampling for the 26% of participants who were asked to provide both), interviewers gave a verbal explanation and leaflet describing the purpose and nature of the saliva test. They explained that the test would be anonymised and that participants would not be told their individual results. Written consent was obtained for collecting and testing the sample and, separately, for storing remaining saliva for possible future testing. Full details of the protocol are provided elsewhere.^15^

Our target was to obtain 4400 saliva samples. Initially, a randomly selected 30% of participants aged 18–34 years and 66% of those aged 35–74 years (covering all PSUs) were asked to provide a saliva sample. These proportions were increased during fieldwork to 75% and 100%, respectively. Overall, 9170 eligible participants were asked to provide a sample, and 6515 agreed to do so (71.0%). Since the equipment was left with participants to provide samples the morning after the interview, further dropout was expected at this stage. Samples were received from 4591 participants (70.5% of those who agreed and 50.1% of eligible participants). After discarding samples that could not be used (eg, because of insufficient volume), the number of useable samples was 4128 (45.0% of eligible participants).

To minimise the effect of diurnal variation in testosterone, participants were asked to provide a saliva sample before 10:00 h. They were asked not to brush their teeth, eat or chew before giving the sample, to reduce potential blood contamination. Participants were asked to drool directly into a polystyrene tube and to post the sample on the day of collection to the Department of Clinical Biochemistry, Glasgow Royal Infirmary (GRI), for processing and storage. Once the saliva samples were received by the laboratory, participants were sent an additional £5 gift voucher.

Samples were prepared at the GRI and sent in batches for testing to the Biochemistry Department at University Hospital South Manchester. They were analysed for testosterone using a newly developed and validated liquid chromatography tandem mass spectrometry assay.^17^

## Weighting of survey data

Weighting was carried out in two stages. The first corrected for participants’ unequal probabilities of selection for inclusion in the sample. For this, two sets of weights were applied: the first to correct for the selection of one household at multi-household addresses, and the second to correct for the varying probabilities of selection by number of adults within households, which also corrected for the unequal probabilities of selection by age (ie, the oversampling of young people aged 16–34 years). These corrections were made by applying weights which were inversely proportional to the selection probabilities for the number of households and adults within the eligible age range at each selected address.

The second stage was to adjust for differential non-response by comparing the age, gender and government office region profile of participants (after applying the selection weights) with 2011 census data. After selection weighting, the achieved sample under-represented participants living in London and men and women aged 20–34 years, while it over-represented men aged 55–74 years and women aged 35–54 years. The final weight was calculated as the product of the selection weight and the non-response weight. After trimming one extreme value, the weights were scaled to have a mean of 1 (which gives a weighted sample size equal to the unweighted sample size). The reduction in sampling efficiency due to the weighting, expressed as the effective sample size, was 72.4% for men and 73.0% for women.

In order to reduce possible bias in the urine sample data arising from differential response, an additional non-response weight was calculated specifically for the urine test results. Response to the urine sample was modelled using logistic regression, with the dependent variable indicating whether or not a useable urine sample was provided. Using data available for both responders and non-responders to the urine sample, a range of demographic and behavioural indicators were included as covariates. The non-response weights for the urine sample were calculated, separately for men and women, as the inverse of the model-predicted probability of obtaining a useable urine sample. After trimming two extreme values, the final urine weights were scaled to have a mean of 1. A similar procedure was followed for weighting the achieved saliva sample data. Full descriptions of weighting all stages of the survey are provided elsewhere.^15^

## Representativeness of the natsal-3 sample

Natsal-3 data were weighted to match the British population in terms of gender, age and geographic region. Natsal-3 participants were compared with other reliable data sources to assess their representativeness. The most reliable external source is the 2011 UK population census.

Table 2 compares distributions for Natsal-3 with three variables from the 2011 census (limited to ages 16–74 years in England and Wales only). The Natsal-3 sample shows a close match to the 2011 census figures for England and Wales on the variables included in table 2. In terms of ethnicity, there is a slight under-representation of Asian men and women in Natsal-3; while looking at self-reported general health, it appears that Natsal-3 participants (men especially) are more likely to classify themselves in ‘fair’ health. In terms of marital status, Natsal-3 over-represents participants who are living with their marital spouse or civil partner, and under-represents men and women who are single. The published census figures relate to ‘all usual residents’ who include individuals living in institutions such as care homes. This may explain some of the differences between the census and Natsal-3 (eg, the higher proportion of single people in the census since individuals not living in residential households are more likely to be single).

**Table 2:** Natsal-3 distributions compared with 2011 population census

England and Wales, ages 16–74	Natsal-3			Census benchmarks		
	Men	Women	All	Men	Women	All
Marital status*	%	%	%	%	%	%
Single, never married	38.1	32.3	35.1	41.0	34.3	37.7
Married, living with spouse	50.0	49.4	49.7	46.7	47.1	46.9
Separated/divorced/widowed	11.6	17.7	14.8	12.0	18.3	15.2
Civil partnership, living with partner	0.4	0.6	0.5	0.3	0.2	0.2
Ethnic group*						
White	86.7	86.9	86.8	86.6	86.8	86.7
Mixed	1.7	2.0	1.8	1.6	1.6	1.6
Asian	7.0	5.7	6.4	7.6	7.4	7.5
Black	3.4	4.0	3.7	3.0	3.3	3.2
Other	1.2	1.4	1.3	1.2	0.9	1.0
Self-reported general health*						
Very good/good	81.1	80.7	80.9	82.1	81.3	81.7
Fair	14.9	14.3	14.6	12.5	13.3	12.9
Bad/very bad	3.9	4.9	4.4	5.4	5.3	5.4

*Census data 2011, all usual residents aged 16–74 in England and Wales.

Another comparison can be made with birth statistics, which are based on births registered in Britain. Using data provided by the Office for National Statistics on the number of live births in 2011, we calculated the birth rate for the population in Britain aged 16–74 years as 17.32 per 1000 persons. Calculated on the same basis, the birth rate for Natsal-3 respondents works out as 18.98 per 1000 persons aged 16–74 years, with 95% CIs (17.11–21.04 per thousand) that overlap the population birth rate.

## Conclusions

While surveys on sexual behaviours have been conducted in other countries,18–23 Natsal has been carried out three times at 10-yearly intervals since 1990 and is now, to the best of our knowledge, the largest, repeated, indepth probability sample survey of sexual behaviour in the world. Natsal's high quality data enable a detailed examination of trends in sexual practices and attitudes over this period.

Natsal-3 built on two earlier surveys by: extending the age range to 74 years; introducing or expanding questionnaire topics; developing a psychometrically validated measure for sexual function; expanding the range of STIs tested for in urine; and validating an assay for measuring testosterone in saliva. All Natsals have used high quality methods including personal interview and, for the most sensitive questions, self-completion formats.

**Figure 1:**
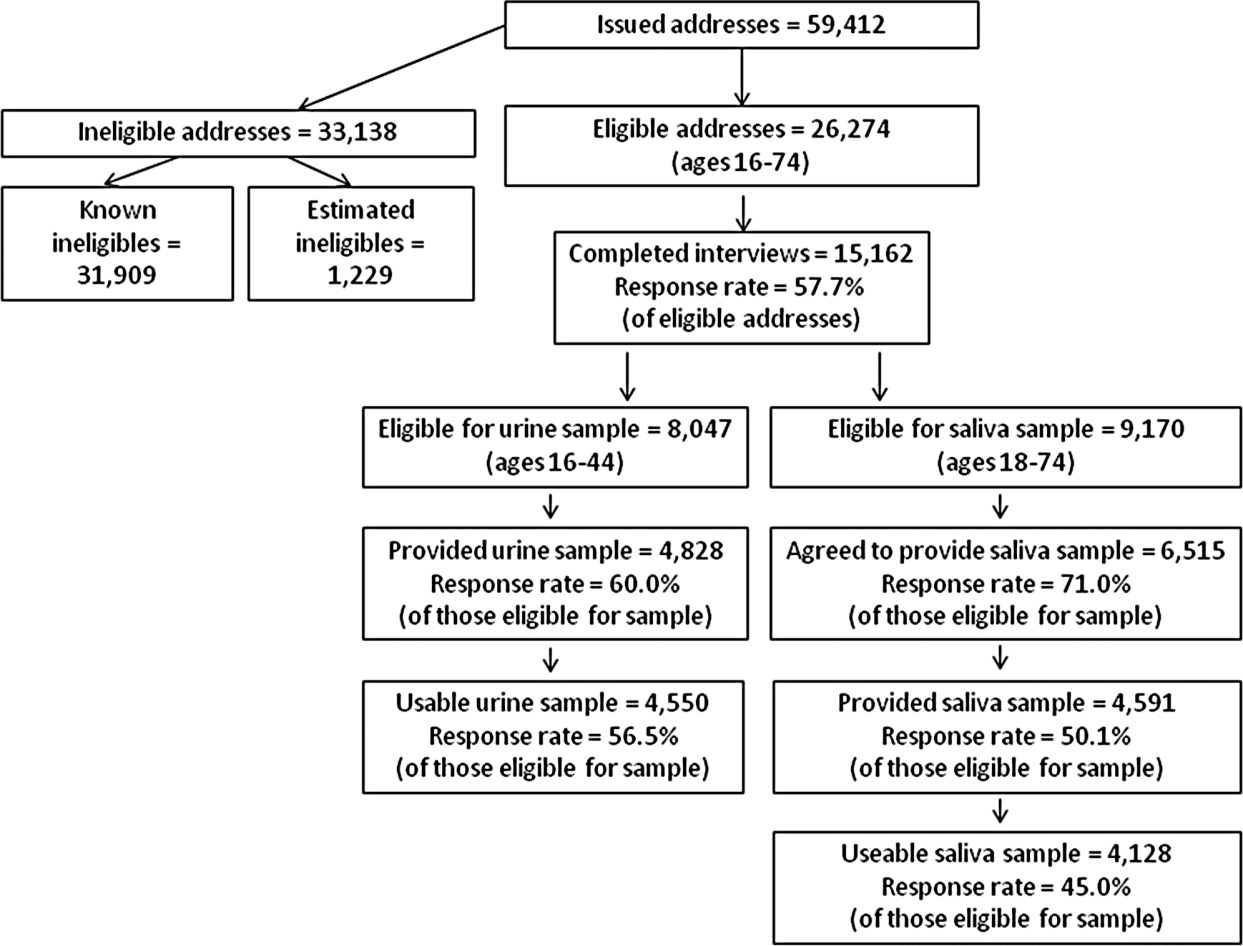
Natsal-3 response summary.

In line with all large-scale face-to-face surveys in Britain, Natsal response has declined over the past 20 years. Several factors explain the lower response achieved in Natsal-3 (57.7%) compared with Natsal-1 (64.7%) and Natsal-2 (65.4%). Survey response rates generally have declined over the last decade;^24^ methods for calculating response rates have changed over time and now provide more conservative estimates; and the different age ranges included in the three surveys affect response, as is apparent from the higher response achieved on the boost samples in Natsal-3. Given that the 64.8% response rate for Natsal-3 ‘boost 1’ is only marginally lower than Natsal-2 response, while covering a similar (albeit narrower) age range (16–34 and 16–44 years, respectively), it appears that Natsal-3 response is comparable with that achieved a decade earlier. Moreover, Natsal-3 response is similar to response on other surveys in Britain, such as the British Social Attitudes Survey.^25^ Figure 1 summarises response to the different elements of Natsal-3.

The dataset was weighted to be representative of the British population in terms of age, gender and region. While bias cannot be ruled out for any survey, comparisons with 2011 census data show that the survey achieved good representation on other characteristics, including marital status, ethnicity and general health.

Natsal-3 provides a rich dataset that should be widely used by researchers, and an anonymised dataset will be deposited with the UK Data Archive, which also holds the previous two survey datasets.

**Key messages**

- Britain's third National Survey of Sexual Attitudes & Lifestyles (Natsal-3) interviewed 15 162 participants aged 16–74 years between 2010 and 2012.
- Natsal-3 used high-quality methods including computer-assisted personal interview and self-interview, along with a multi-stage probability sample design.
- Natsal-3 achieved a response rate of 57.7%, in line with the two previous Natsal surveys and other high profile British social surveys.
- Compared with the earlier surveys, Natsal-3 included a wider age range, new questionnaire topics, more sexually transmitted infections tested in urine and testosterone measured in saliva.

## Supplementary Material

Web supplement
