# Supplementary material for: Methodology of the third British National Survey of Sexual Attitudes and Lifestyles (Natsal-3)
Source: Sex Transm Infect. 2013 Nov 26;90(2):84–9. doi: 10.1136/sextrans-2013-051359 (PMC3933071; doi:10.1136/sextrans-2013-051359)
Supplement: Web supplement [file sextrans-2013-051359-s1.pdf]

Natsal-3 methodology: web only appendix

Fieldwork was split into eight 'waves', with each wave issued roughly every quarter over the two years allocated to data collection. Although the number of issued sectors varied per wave, since they were randomly selected, each wave provides a nationally representative sample of the general population. In order to meet the overall target of 15,000 achieved interviews, as well as the targets for individual age groups, the sample design was 'fine tuned' during the two year period of fieldwork. A summary of the number of PSUs and addresses issued per wave is found in Table A1.

Web Table A1 Number of PSUs and addresses issued per wave of fieldwork by sample type

	**PSUs issued**	**Addresses/PSU**			
		**Total**	**Core (16-74)**	**Boost 1 (16-34)**	**Boost 2 (16-29)**
Wave 1<br>Sep–Dec 2010	225	30	12	10	8
Wave 2<br>Jan–Mar 2011	235	30	12	10	8
Wave 3<br>Apr–Jun 2011	217	36	15	11	10
Wave 4<br>Jul–Sep 2011	217	36	15	11	10
Wave 5<br>Oct–Dec 2011	217	36	15	11	10
Wave 6<br>Jan–Mar 2012	217	36	15	11	10
Wave 7<br>Mar–May 2012	217	36	16	11	9
Wave 8<br>May–Aug 2012	182	36	16	11	9
**TOTAL PSUs/ addresses**	**1727**	**59412**	**24924**	**18537**	**15951**
